# Clear Cell Renal Cell Carcinoma with Synchronous Bladder Metastasis: Diagnostic, Surgical, and Pathological Insights from a Rare Presentation

**DOI:** 10.3390/jcm15062098

**Published:** 2026-03-10

**Authors:** Miroslava Benkova-Petrova, Alexander Petrov, Pavel Abushev, Plamen Kirilov, Simeon Marinov, Doroteya Malinova, Stanila Stoeva-Grigorova

**Affiliations:** 1 Clinic of Nephrology, University Hospital “St. Marina”, 9010 Varna, Bulgaria; miroslava.benkova@mu-varna.bg (M.B.-P.); alexander.petrov1@gmail.com (A.P.); pavel.abushev@mu-varna.bg (P.A.); 2 Department of Urology, Faculty of Medicine, Medical University of Varna, 9000 Varna, Bulgaria; plamen.kirilov@mu-varna.bg (P.K.); dr.marinov.simeon95@gmail.com (S.M.); doroteya.malinova@mu-varna.bg (D.M.); 3 Clinic of Urology, University Hospital “St. Marina”, 9010 Varna, Bulgaria; 4 Clinic of General and Clinical Pathology, University Hospital “St. Marina”, 9010 Varna, Bulgaria; 5 Department of Pharmacology, Toxicology and Pharmacotherapy, Faculty of Pharmacy, Medical University of Varna, 9000 Varna, Bulgaria

**Keywords:** clear cell renal cell carcinoma, bladder metastasis, transurethral resection, robotic nephro-adrenalectomy, Immunohistochemistry, lymphovascular invasion, multidisciplinary management, immune checkpoint inhibitors, tyrosine kinase inhibitors, metastatic RCC

## Abstract

**Background:** Clear cell renal cell carcinoma (ccRCC) constitutes 75–80% of all renal cell carcinomas and exhibits aggressive behavior with high metastatic potential. Common metastatic sites include lungs, bones, lymph nodes, and liver, while urinary bladder involvement is exceedingly rare. Early detection of atypical metastases is critical for risk stratification, surgical planning, and systemic therapy selection. **Methods:** We report a 69-year-old male presenting with recurrent, painless gross hematuria and dysuria. Contrast-enhanced computed tomography revealed a left renal mass with bilateral pulmonary nodules, regional lymphadenopathy, and a bladder lesion. The patient underwent transurethral resection (TUR) of the bladder lesion, followed by robot-assisted left nephro-adrenalectomy with para-aortic lymphadenectomy. Histopathology and immunohistochemistry (PAX8+, CD10+, CAIX+, CK7−, GATA3−) confirmed ccRCC with synchronous bladder metastasis. Postoperatively, combined immune checkpoint inhibitor (ICI) therapy and tyrosine kinase inhibitors (TKIs) were initiated. **Results:** TUR provided symptomatic relief and diagnostic confirmation. Robot-assisted surgery enabled precise, oncologically safe excision of the primary tumor and regional metastases with minimal blood loss and no perioperative complications. Pathological staging was pT3aN1M1, ISUP grade 2, with lymphovascular invasion, confirming advanced disease requiring systemic therapy. Early initiation of ICI plus TKI therapy targeted residual micrometastases to potentially prolong survival. **Conclusions:** This case highlights the rare occurrence of ccRCC with synchronous bladder metastasis and underscores the importance of comprehensive imaging, detailed morphologic and immunohistochemical evaluation, and a multidisciplinary approach. Robot-assisted cytoreductive surgery combined with modern systemic therapy represents an effective strategy for advanced ccRCC, emphasizing the need for individualized treatment and long-term follow-up in atypical metastatic scenarios.

## 1. Introduction

Renal cell carcinoma (RCC) accounts for approximately 2–3% of all adult malignancies and remains one of the most aggressive urologic cancers. The disease is most commonly diagnosed between 60 and 70 years of age, although it may also occur in younger patients. Men are disproportionately affected, with a male-to-female ratio of approximately 1.5–2:1 [1]. The most common histological subtype is clear cell renal cell carcinoma (ccRCC), accounting for approximately 75–80% of all RCC cases. This predominance underscores its clinical significance, with important implications for diagnosis, therapeutic decision-making, and prognostic assessment [2,3,4,5,6]. ccRCC is characterized by a more aggressive biological behavior and poorer prognosis compared to other subtypes, such as papillary or chromophobe RCC [7,8]. These observations highlight that timely identification of ccRCC is critical for accurate risk stratification, informed decisions between surgical intervention and active surveillance, and the optimal selection of systemic, targeted, or immunotherapeutic strategies [4,9,10].

The genetic hallmark of ccRCC, most commonly involving alterations in the VHL gene, represents a key driver of tumor development, leading to pronounced vascularization and an elevated metastatic potential [11]. Despite advances in imaging and modern early detection strategies, approximately 20–30% of patients are diagnosed with metastatic disease at initial presentation [12]. This is largely attributable to the frequently asymptomatic nature of early-stage RCC, with clinical manifestations becoming apparent only after regional or distant dissemination has occurred [13]. Metastases in ccRCC most commonly involve the lungs (~45% of cases), bones (~30%), lymph nodes (~22%), and liver (~20%), reflecting the aggressive behavior of the disease [14,15,16]. In contrast, metastasis to the urinary bladder is exceedingly rare, reported in approximately 2% of cases, with some studies documenting only a few dozen instances worldwide [17,18,19]. The rarity of bladder involvement is largely explained by the anatomical organization of the venous circulation and the relatively “isolated” pathway of the urinary tract. Several mechanisms have been proposed, including retrograde venous flow through the renal and gonadal venous plexuses, lymphatic reflux, and hematogenous microembolization [20]. In select cases, tumor cell implantation via urinary “shedding” has also been suggested, particularly when synchronous involvement of the ureter or bladder is present [21,22]. Clinically, such metastases often present with gross hematuria, which can mimic primary urothelial carcinoma and potentially delay diagnosis [17].

The present study aims to document an exceptionally rare case of ccRCC with synchronous bladder metastasis and to provide a comprehensive analysis of the diagnostic, surgical, and pathological aspects of such a presentation. The report integrates detailed imaging findings, histopathological and immunohistochemical assessments, as well as the operative strategy involving robot-assisted nephro-adrenalectomy with lymphadenectomy, illustrating a contemporary multidisciplinary approach to the management of advanced ccRCC. The novelty of this manuscript lies in its systematic presentation of the rare phenomenon of bladder metastasis, supported by clinical, pathological, and therapeutic observations, thereby expanding current knowledge on ccRCC biology and offering practical guidance for the recognition, differentiation, and individualized management of these patients. This case underscores the importance of long-term follow-up and careful identification of atypical metastatic sites to optimize prognosis and therapeutic outcomes in high-risk RCC patients.

## 2. Case Presentation

### 2.1. Patient Information

The patient is a 69-year-old male who was admitted to the Department of Nephrology and Urology at St. Marina University Hospital—Varna in October 2025, presenting with recurrent, painless gross hematuria and mild dysuria of approximately two weeks’ duration. His past medical history is notable for type 2 diabetes mellitus, arterial hypertension, and stage 2 chronic kidney disease according to KDIGO criteria. The patient reported no occupational exposures, is a non-smoker, and has no family history of malignancy.

### 2.2. Clinical Findings

Upon admission, the patient was hemodynamically stable and afebrile. Physical examination revealed normal cardiovascular and respiratory findings. The abdomen was soft and non-tender, without palpable renal masses or organomegaly. Laboratory tests showed mild normocytic anemia, slightly elevated serum creatinine, and borderline high urea. Urinalysis revealed significant hematuria, mild proteinuria, and glucosuria. The key laboratory results are summarized in Table 1.

### 2.3. Diagnostic Assessment and Treatment Procedures

#### 2.3.1. Imaging

**Figure 1:**
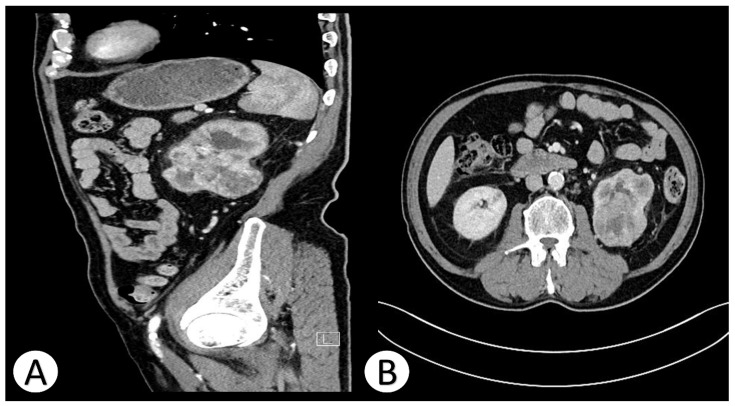
Contrast-enhanced CT of the patient’s abdomen, shown in sagittal (**A**) and axial (**B**) views (the white letter “L” denotes the left side).

Contrast-enhanced computed tomography (CT) of the abdomen revealed a large (51 × 61 × 52 mm), heterogeneous, well-circumscribed mass originating from the mid to lower pole of the left kidney. The lesion exhibited mixed density with solid and necrotic components and heterogeneous contrast enhancement, consistent with a hypervascular renal tumor. Multiple bilateral pulmonary nodules (up to 24 mm) were also identified, highly suspicious for metastatic deposits. Additionally, para-aortic and paracaval lymphadenopathy (up to 18 mm) was visualized (Figure 1).

For distant staging, a whole-body PET-CT scan was performed (50 min post-injection of 18F-FDG, 139 MBq). Bilateral pulmonary nodules were confirmed as multiple scattered parenchymal lesions up to 32 × 18 mm, with a maximum SUV of 9.61, consistent with metastatic deposits. No cavitation was observed. No hypermetabolic mediastinal, hilar, or axillary lymph nodes were detected. Three hypermetabolic right paraaortocaval lymph nodes were noted (up to 18 × 12 mm, SUVmax 3.38). The left kidney showed status post-nephrectomy with no evidence of local recurrence, while the previously resected bladder lesion corresponded to metastatic ccRCC. Additional hypermetabolic foci were identified in skeletal muscles (two in the left arm, one in the right arm, and one in the gluteal region; SUVmax 3.1–3.8), suspected for secondary involvement. No hypermetabolic lesions were observed in the brain, pelvic organs, bones, or inguinal lymph nodes.

#### 2.3.2. Transurethral Resection (TUR)

Due to marked bladder wall thickening and the presence of hematuria, the patient underwent TUR of a 4 cm papillary lesion located at the bladder dome in early October 2025. Intraoperatively, the lesion appeared highly vascularized with a yellowish hue. The resection was complete, and hemostasis was adequately achieved. The excised tissue was submitted for histopathological and immunohistochemical analysis.

#### 2.3.3. Histopathology and Immunohistochemistry (Post-TUR)

**Figure 2:**
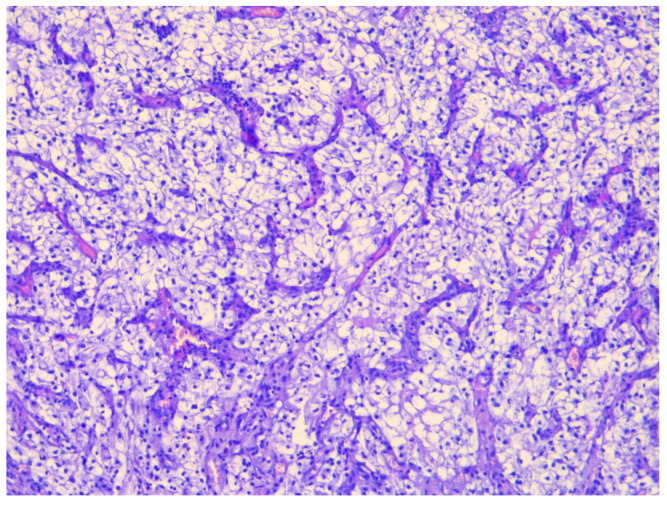
Histopathological image of a metastatic ccRCC lesion in the urinary bladder, demonstrating alveolar architecture, prominent vascularization, and marked cellular atypia (clear cytoplasm, round nuclei, ×100).

Histopathological examination revealed nests and alveolar arrangements of atypical clear cells with optically empty cytoplasm, well-defined cell borders, and hyperchromatic nuclei, organized around thin-walled, branching blood vessels. Foci of necrosis and hemorrhage were also observed. These morphological features were consistent with metastatic ccRCC, confirming secondary involvement of the urinary bladder from a primary renal tumor (Figure 2).

Immunohistochemical profiling further corroborated the diagnosis of metastatic ccRCC with bladder involvement. Tumor cells exhibited positive staining for PAX8, CD10, and CAIX, and were negative for CK7 and GATA3. This immunophenotype is characteristic of ccRCC and allows clear distinction from primary urothelial carcinoma [23,24]. These findings underscore the critical importance of integrating detailed morphological assessment with immunohistochemical verification for the accurate identification of extrarenal metastatic sites of ccRCC and for guiding informed decisions regarding optimal therapeutic strategies.

#### 2.3.4. Robot-Assisted Left Nephro-Adrenalectomy with Para-Aortic Lymphadenectomy

The patient underwent a planned radical robot-assisted left nephro-adrenalectomy with para-aortic lymphadenectomy later in October 2025, using the Da Vinci Xi surgical platform. This approach enabled a minimally invasive, precise, and oncologically safe excision of a large renal mass, including the adjacent adrenal gland. Intraoperatively, the left kidney exhibited marked hypertrophy (21 × 13 × 10 cm) and contained a nodular, yellow-ochre tumor mass measuring 7.5 cm, infiltrating the renal hilum and surrounding perirenal fat. The adrenal gland was resected en bloc with the primary tumor. No peritoneal deposits or other intra-abdominal abnormalities were identified. The procedure was completed in 80 min without intraoperative complications. Hemodynamic parameters remained stable throughout, and the estimated blood loss was 200 mL.

A formal lymphadenectomy was not performed, as it is generally not indicated in patients presenting with distant metastases. The surgical margin distance of the primary tumor was >5 mm. The adrenal involvement was contiguous with the primary renal tumor rather than representing a separate metastatic focus.

#### 2.3.5. Histopathology Following Nephro-Adrenalectomy

Histopathological analysis confirmed the diagnosis of ccRCC, staged as pT3aN1M1, ISUP grade 2, with evidence of lymphovascular invasion (LVI+). The tumor cells exhibited classic clear cytoplasm and delicate vascular architecture characteristic of ccRCC, correlating with a high potential for both local and distant metastasis.

**Figure 3:**
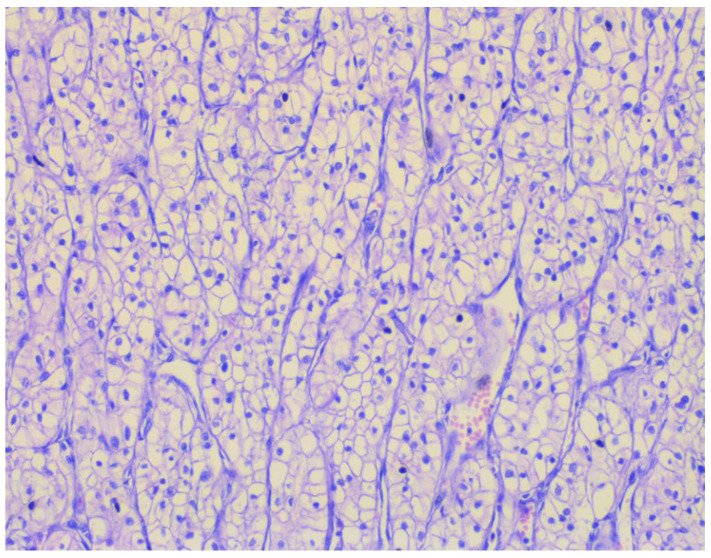
Representative histology of surgically resected ccRCC (pT3aN1M1, ISUP grade 2) showing classical clear cytoplasm, fine vascular network, and lymphovascular invasion.

Metastatic involvement of the adrenal gland and para-aortic lymph nodes was confirmed, establishing stage IV disease and indicating the need for ongoing systemic therapy [25,26]. Notably, the surgical resection margins, ureter, and hilar vessels remained free of tumor infiltration, demonstrating complete oncologic excision of the primary lesion and regional structures (Figure 3). These findings underscore the critical importance of combining radical surgical resection with precise histopathological assessment for accurate oncologic staging and prognosis, as well as for the strategic planning of subsequent systemic therapy in patients with metastatic ccRCC.

### 2.4. Follow-Up and Outcomes

The postoperative course was uneventful, with the patient remaining afebrile, maintaining satisfactory urine output and stable renal function (serum creatinine: 111 µmol/L), and showing no evidence of hemodynamic instability or perioperative complications. Five days following the robot-assisted procedure, the patient was discharged with clear instructions for regular follow-up and initiation of systemic oncologic therapy.

The standard therapeutic regimen comprised a combination of an immune checkpoint inhibitor (ICI) and a tyrosine kinase inhibitor (TKI), a strategy that has demonstrated efficacy in patients with stage IV metastatic ccRCC. According to the International Metastatic Renal Cell Carcinoma Database Consortium (IMDC) criteria, the patient was classified as intermediate risk (IMDC score: 3), which further supported the decision to initiate combination systemic therapy [27].

Oncologic follow-up is ongoing. A PET-CT scan and a follow-up cystoscopy are scheduled at three months after the surgical procedure. At the time of manuscript submission, the patient’s postoperative follow-up period was limited, and early radiographic response has not yet been assessed.

## 3. Discussion

This clinical case illustrates an exceptionally rare presentation of metastatic ccRCC with secondary involvement of the urinary bladder. Such occurrences are reported in only a small proportion of patients and are largely limited to isolated case reports and literature reviews, underscoring the importance of documenting these cases [28]. Management of these patients requires careful consideration of the diagnostic challenges in differentiating metastatic lesions from primary urothelial carcinoma, as clinical symptoms and imaging findings frequently overlap. This is particularly relevant in the context of gross hematuria, with or without irritative lower urinary tract symptoms, which was also observed in our patient. Imaging findings may reveal solitary or multifocal bladder wall thickenings that are indistinguishable from primary bladder carcinoma. In this context, accurate identification of the metastatic origin of the bladder lesion through combined morphologic assessment and immunohistochemical profiling (PAX8+, CD10+, CAIX+, CK7−, GATA3−) was critical to confirm metastatic ccRCC and exclude primary urothelial carcinoma. These immunohistochemical markers have been previously described as highly specific and clinically valuable for differentiating ccRCC from urothelial tumors [29].

The mechanism of bladder metastasis in ccRCC remains incompletely understood, with proposed pathways including retrograde venous flow, lymphatic spread, hematogenous microembolization, and intraluminal tumor shedding. In this case, the overall metastatic pattern strongly supports systemic hematogenous dissemination rather than urinary shedding. The presence of bilateral pulmonary metastases, paraaortic nodal involvement (N1), adrenal metastasis, and confirmed lymphovascular invasion (LVI+) collectively indicate established systemic vascular dissemination [17,30,31]. No direct invasion of the renal pelvis or collecting system was observed, making intraluminal implantation unlikely. While urinary seeding has been reported in rare cases, it is usually associated with collecting system involvement or tumor thrombus in the ureter. Therefore, given the synchronous distant metastases, hematogenous spread represents the most biologically plausible mechanism in this patient [17,20].

In this case, the decision to perform radical robot-assisted nephro-adrenalectomy with para-aortic lymphadenectomy using the Da Vinci Xi platform reflects a contemporary multidisciplinary approach to locally advanced urologic malignancies. Robotic surgery has become one of the most widely employed minimally invasive techniques in urology for the management of renal malignancies, including RCC, owing to enhanced three-dimensional visualization, articulated instruments with extensive freedom of movement, and tremor filtration, which collectively facilitate precise oncologic resection [32,33,34,35,36,37,38,39]. Reduced intraoperative blood loss and expedited recovery are particularly important for patients with large or locally advanced tumors, as in the present case, minimizing surgical stress and early postoperative complications [37]. Furthermore, the use of robotic systems markedly enhances intraoperative visualization and dissection precision, enabling surgeons to operate confidently within confined anatomical spaces while achieving negative resection margins without increasing the incidence of positive surgical margins—a critical prognostic determinant in oncologic procedures. These attributes render robotic technology particularly well-suited for complex urologic interventions, including nephron-sparing approaches such as partial nephrectomy, where preservation of renal parenchyma is essential [32]. It is perhaps for this reason that, in a forward-looking perspective, some authors argue that the advent of minimally invasive surgery in the robotic era necessitates that comparative analyses be conducted not merely between open and laparoscopic approaches, but among the various robotic platforms themselves [40]. Notwithstanding these advantages, limitations include the absence of direct tactile feedback, longer operative duration, and higher procedural costs [36,41,42]. Although short-term outcomes—such as reduced blood loss and shorter hospitalization—are consistently favorable, robust evidence demonstrating superior long-term survival compared with open or laparoscopic surgery remains limited. This underscores the necessity for meticulous patient selection and specialized surgical team training [34,43,44,45]. We concur with this perspective and emphasize that the generation of more compelling evidence requires broader documentation and dissemination of comparable cases, particularly in the context of rare clinical presentations.

In addition to surgical interventions, patients with widely metastatic RCC may be considered for non-surgical or palliative strategies, particularly when tumor burden is high or significant comorbidities exist. Such approaches include local ablation, palliative radiotherapy for symptom control, and systemic therapy when complete resection is not feasible [46,47,48]. Radiotherapy has demonstrated efficacy in alleviating pain and controlling local symptoms, especially for bone lesions or nerve compression [49]. Furthermore, in patients with high tumor burden who are not candidates for aggressive surgery, systemic therapy with ICI, TKI, or their combination can serve as the main therapeutic strategy, providing disease control and improving quality of life [50,51]. In the present case, given the patient’s relatively good general condition, absence of significant organ dysfunction, and the feasibility of complete surgical resection, cytoreductive surgery was deemed justified prior to initiation of systemic therapy, allowing tumor burden reduction before immunotherapy and targeted therapy.

Despite complete surgical resection, the presence of pulmonary, lymphatic, and adrenal metastases indicated stage IV disease, warranting systemic therapy. Management of metastatic RCC has shifted with the introduction of ICI-based therapies, alone or combined with TKIs, which enhance antitumor immunity and improve outcomes [52]. Phase III trials and meta-analyses support ICI + TKI combinations as first-line therapy, showing superior progression-free and overall survival compared with sunitinib, including regimens such as nivolumab + cabozantinib, pembrolizumab + lenvatinib, pembrolizumab + axitinib, avelumab + axitinib, and nivolumab + ipilimumab [53]. Clinical registry data indicate >80% overall survival at 12 months, median progression-free survival of 19 months, and ORR of ~69% [54]. While efficacy may decline in later lines, these regimens remain safe and feasible, with ongoing evaluation of predictive biomarkers and manageable toxicity profiles [55,56]. Overall, evidence from trials and real-world cohorts confirms ICI + TKI as an effective, safe first-line option, emphasizing multidisciplinary management [57,58,59,60,61,62,63].

Against the backdrop of the proven benefits of contemporary immuno-oncologic strategies, clinical outcomes in patients with advanced and metastatic disease remain highly heterogeneous, largely dependent on tumor burden, the intrinsic biological characteristics of the neoplasm, and individual responses to systemic therapy. Literature data indicate that the median survival following the diagnosis of bladder metastases ranges from 6 to 30 months, underscoring the aggressive nature of the disease and the critical need to optimize therapeutic strategies [64]. In this context, the present clinical case, together with an analysis of current evidence, highlights that early detection of metastatic spread, cytoreductive surgery when oncologically justified, and the implementation of modern systemic therapies constitute fundamental and mutually complementary components of comprehensive patient management. Such a multimodal and individualized approach not only provides the potential to prolong overall survival but also contributes to improved disease control, symptom alleviation, and enhanced quality of life. These objectives remain the paramount goals in the management of patients with advanced RCC.

Although this clinical case provides detailed diagnostic, surgical, and therapeutic information on a rare metastatic presentation of ccRCC, it has inherent limitations. As a single-case report, the findings cannot be generalized to a broader population, and the observed therapeutic effects are descriptive, lacking a control group. Furthermore, follow-up at the time of manuscript submission is limited, and long-term data on survival and response to systemic therapy are not yet available. These limitations should be considered when interpreting the results and underscore the need for larger cohort studies and prospective investigations to validate the observations reported here.

## 4. Conclusions

The presented clinical case illustrates an exceptionally rare and clinically challenging presentation of ccRCC with synchronous bladder metastasis, highlighting the complexity of both diagnostic and therapeutic management in advanced disease. The atypical metastatic site, clinically manifesting as gross hematuria, necessitated an integrated, multimodal diagnostic approach, encompassing advanced imaging, detailed morphological assessment, and extensive immunohistochemical profiling to reliably distinguish metastatic ccRCC from primary urothelial carcinoma.

This case underscores the pivotal role of cytoreductive surgery, performed via robot-assisted nephro-adrenalectomy with lymphadenectomy, as part of a contemporary multidisciplinary framework for managing locally advanced and metastatic ccRCC. The Da Vinci Xi robotic platform enabled precise and oncologically adequate resection of the primary tumor and regional metastatic structures with minimal operative morbidity, thereby optimizing conditions for subsequent systemic therapy. Despite complete surgical excision, the presence of pulmonary, lymphatic, and adrenal metastases defined stage IV disease and warranted initiation of systemic therapy. Current evidence strongly supports the use of immune checkpoint inhibitors, alone or in combination with tyrosine kinase inhibitors, as first-line therapy in metastatic RCC, demonstrating significant improvements in both overall and progression-free survival. Nevertheless, clinical prognosis remains heterogeneous, particularly in patients with rare metastatic sites such as the bladder, emphasizing the importance of individualized therapeutic strategies.

In conclusion, this case highlights the necessity for heightened clinical vigilance, long-term follow-up, and interdisciplinary collaboration in ccRCC management. Furthermore, the documentation and publication of rare clinical presentations are critical to expanding understanding of tumor biology and behavior. A multimodal, personalized approach represents a cornerstone for optimizing oncological control, prolonging survival, and enhancing quality of life in patients with advanced RCC.

## Figures and Tables

**Table 1:** Laboratory findings at admission.

Parameter	Result	Reference Range	Interpretation
Hemoglobin	117 g/L	125–172 g/L	Mild normocytic anemia
Serum creatinine	118 µmol/L	62–115 µmol/L	Slightly elevated
Urea	8.6 mmol/L	3.2–8.2 mmol/L	Mildly elevated
Urinalysis: Hematuria	3+	Negative	Significant hematuria
Urinalysis: Proteinuria	1+	Negative	Mild proteinuria
Urinalysis: Glucosuria	2+	Negative	Mild glucosuria

## Data Availability

No new datasets were generated or analyzed during the preparation of this case report.

## Abbreviations

The following abbreviations are used in this manuscript:

ccRCC	Clear cell renal cell carcinoma
CT	Computed tomography
IMDC	International Metastatic Renal Cell Carcinoma Database Consortium
ICI	Immune checkpoint inhibitors
PD-1	Programmed Death-1
PD-L1	Programmed Death-Ligand 1
RCC	Renal cell carcinoma
TKI	Tyrosine kinase inhibitor
TUR	Transurethral resection
